# Case Report: Treatment of a Comorbid Attention Deficit Hyperactivity Disorder and Obsessive–Compulsive Disorder With Psychostimulants

**DOI:** 10.3389/fpsyt.2021.649833

**Published:** 2021-05-12

**Authors:** Ezgi Dogan-Sander, Maria Strauß

**Affiliations:** Department of Psychiatry and Psychotherapy, University of Leipzig Medical Center, Leipzig, Germany

**Keywords:** ADHD, OCD, comorbidity, psychostimulant, methylphenidate, case report

## Abstract

**Introduction:** Attention deficit hyperactivity disorder (ADHD) is a common disease in childhood and adolescence. In about 60% of pediatric patients, the symptoms persist into adulthood. Treatment guidelines for adult ADHD patients suggest multimodal therapy consisting of psychostimulants and psychotherapy. Many adult ADHD patients also suffer from psychiatric comorbidities, among others obsessive–compulsive disorder (OCD). The treatment of the comorbidity of ADHD and OCD remains challenging as the literature is sparse. Moreover, the impact of psychostimulants on obsessive–compulsive symptoms is still unclear.

**Case Presentation:** Here, we report on a 33-year-old patient with an OCD who was unable to achieve sufficient remission under long-term guideline-based treatment for OCD. The re-examination of the psychological symptoms revealed the presence of adult ADHD as a comorbid disorder. The patient has already been treated with paroxetine and quetiapine for the OCD. Due to the newly established diagnosis of ADHD, extended-release methylphenidate (ER MPH) was administered in addition to a serotonin reuptake inhibitor. After a dose of 30 mg ER MPH, the patient reported an improvement in both the ADHD and the obsessive–compulsive symptoms. After discharge, the patient reduced ER MPH without consultation with a physician due to subjectively described side effects. The discontinuation of medication led to a renewed increase in ADHD and obsessive–compulsive symptoms. The readjustment to ER MPH in combination with sertraline and quetiapine thereafter led to a significant improvement in the compulsive symptoms again.

**Conclusion:** The present case shows that in ADHD and comorbid obsessive–compulsive disorder, treatment with psychostimulants can improve the obsessive–compulsive symptoms in addition to the ADHD-specific symptoms. To our knowledge, this is only the second case report describing a treatment with ER MPH for an adult patient with OCD and ADHD comorbidity in the literature. Further research, especially randomized controlled trials, is needed to standardize treatment options.

## Introduction

Attention deficit hyperactivity disorder (ADHD) is a frequent mental disorder with childhood onset and a worldwide prevalence of at least 2.8% (1). It is characterized by the three core symptoms of attention deficit, hyperactivity, and impulsivity manifesting since childhood (2). Adult ADHD is also commonly associated with different comorbidities (3, 4), particularly obsessive–compulsive disorder (OCD). The prevalence of OCD comorbidity in patients with ADHD varies widely in the literature, ranging from 1 to 13% (5). On the other hand, ADHD prevalence in patients with OCD has been reported as ranging from 0 to 23% (5). The high co-occurrence of these disorders has raised questions about their diagnoses, neurobiology, and treatment.

It has been discussed that the ADHD-like symptoms in OCD, for example inattention, may have contributed to the inconsistency of the reported co-occurrence rates. Furthermore, familial link between OCD and ADHD, disturbances in attention, and executive function and the high comorbidity of tic disorders are common features of these two disorders (6–9).

On the other hand, these disorders have reverse fronto-striatal abnormalities (5). OCD patients exhibit increased fronto-striatal activity and functional connectivity (5). In contrast, ADHD is found to be associated with hypoactivity in the prefrontal and striatal brain regions and a reduced fronto-striatal activity (5). Despite these differences, a shared dysfunction in the medio-fronto-striato-limbic brain region was reported in addition to disorder-specific dysfunctions (10).

Psychostimulants such as methylphenidate are regarded as the first-line treatment for ADHD. They increase prefrontal activation and improve both clinical symptomology and neurocognitive functioning in ADHD by modulating dopamine reuptake. Guidelines for the treatment of OCD recommend serotonin reuptake inhibitors as first-line pharmacotherapy, which are thought to modulate fronto-striatal hyperactivity. In the case of partial response to serotonin reuptake inhibitors, an augmentation therapy with antipsychotics has also been shown to have a useful effect (11).

Although the pharmacotherapy of each of these disorders has been well-established, the effective treatment and management of patients with comorbid ADHD and OCD remains challenging. While stimulant medication is recommended as the first-line treatment for ADHD, findings suggest that its use in OCD may exacerbate the OCD symptoms. To our knowledge, there have been only a few studies, mostly case reports and case studies, reporting on the pharmacotherapy of this comorbidity. Some of these reports have shown that the use of stimulants may cause obsessive–compulsive symptoms as side effects (12–14), while others have reported a decline of OCD symptoms under stimulant therapy (15, 16).

In this report, we present a case of an adult patient with comorbid ADHD and OCD treated successfully with stimulants and serotonin reuptake inhibitors.

## Case Presentation

In November 2017, a 33-year-old patient presented at our ADHD outpatient clinic in the Department of Psychiatry and Psychotherapy at the University Hospital of Leipzig for diagnostic clarification. During a previous psychiatric examination organized by the federal employment agency, a tentative ADHD diagnosis was made for the first time. The patient reported impulsiveness and physical restlessness that had persisted since childhood. He stated that he could hardly sit still or stay in one place for a longer period of time. He also described a lack of concentration and problems sustaining attention in given tasks (see Table 1 for the summary of clinical manifestations). In order to relax physically, he started practicing martial arts and has been doing a lot of gardening lately.

**Table 1:** Summary of the clinical manifestations of ADHD and OCD.

	**Age onset**	**Clinical manifestation**
ADHD	Unknown, probably primary school age	Inattention: easily distracted, forgetful, difficulty in organizing tasks and activities, difficulty in sustaining attention Hyperactivity and impulsiveness: difficulty in waiting for his turn, restlessness, difficulty to remain seated, excessive talking
OCD	10 years	Obsessive thoughts: fear of aliens and the special meaning of the color “blue” because of its association to aliens Obsessive slowness: impaired function and lack of concentration due to obsessive thoughts and compulsive behavior Compulsion: counting and ritualized touching

*ADHD, attention deficit hyperactivity disorder; OCD, obsessive–compulsive disorder*.

A mental status examination was conducted according to the AMDP System (17). The patient was oriented with regard to time, place, person, and situation. He was friendly and cooperative in personal contact. In motor activity, he demonstrated restlessness (fidgeting with the legs, playing with the fingers, and partly increased body tension). He described his mood as slightly dysphoric; his affect was broad. He showed no evidence of delusions, hallucinations, or ideas of reference, but he had poor impulse control, attention deficits with quick distractibility, as well as concentration and short-term memory problems. The thought process was lightly circumstantial, but apart from that without a pathological finding. He did not display any sleep or eating disorders. Any kind of suicidal ideations were denied. The patient demonstrated insight into his mental disorder and was motivated for therapy. These aspects were also confirmed by a senior psychiatrist.

In further exploration, the patient stated that he had been suffering from an OCD since about the age of 10. At that time, a classmate had had an eye tumor, and in this context, he had first developed a washing compulsion for which a first presentation to a psychiatrist had taken place. Later on, he showed compulsive behavior in the form of compulsive counting and ritualized touching things and obsessive thoughts (fear of aliens and the special meaning of the color “blue”). These obsessions began after he watched a film about aliens as a teenager, which frightened him enormously although he does not believe in aliens. Overall, obsessive and compulsive symptoms have been affecting his life in many ways, but especially his work life, disrupting his functionality. He had been treated as an inpatient and outpatient several times, yet the OCD symptoms would still occupy 3–4 h per day (see Table 1). In addition, ambulatory psychotherapy (anamnestically cognitive behavioral therapy) had only helped him to a limited extent. However, the existing concentration problems were described as independent of obsessive–compulsive disorder. The current medication at the first visit consisted of paroxetine 30 mg/day and quetiapine 100 mg/day.

The patient also reported that, in the past, he had been drinking a lot of alcohol to compensate for his compulsions and impulsiveness. However, alcohol had disinhibited him in parts even more, and it had come to physical confrontations several times. He had lost control in situations in which he felt provoked. In the past, criminal proceedings had also been brought against him in this context. In the course of time, he developed an alcohol addiction. At the time of the first visit to our outpatient clinic, he had been completely abstinent from alcohol for 6 years. Drug consumption was also negated, which could also be confirmed by a toxicological screen at the inpatient admission.

The following information was gathered on the past psychiatric history: a first inpatient treatment because of the OCD (ICD-10: F42.2) took place in 2006. During that time, a suspected diagnosis of paranoid schizophrenia (ICD-10: F20.0) was made and treatment with risperidone 1.5 mg/day, olanzapine 10 mg/day, and lorazepam 1 mg/day was started. Risperidone was discontinued due to akathisia, and the patient was then treated with olanzapine 10 mg/day and paroxetine 20 mg/day. In 2008, the patient was treated in a day clinic for 1.5 months, where an OCD (ICD-10: F42.2) and an immature personality accentuation were diagnosed. During this treatment, the dose of sulpride was increased from 200 to 400 mg/day, which was prescribed during the outpatient treatment. Subsequently, sulpride was switched to paroxetine 60 mg/day. In 2009, the patient was hospitalized again due to worsening of the OCD symptoms. In 2012, an alcohol withdrawal treatment was completed. The discharge medication consisted of paroxetine 60 mg/day and olanzapine 10 mg/day. The diagnoses then consisted of alcohol dependence (ICD-10: F10.2), alcohol withdrawal syndrome (ICD-10: F10.3), OCD (ICD-10: F42.2), personality accentuation (ICD-10: F60.9), and an unspecified form of schizophrenia (ICD-10: F20.8). In 2013, another alcohol withdrawal treatment due to a relapse followed. Since then, he has been abstinent of alcohol according to his own statement. Discharge medication consisted of paroxetine 60 mg/day and promethazine 25 mg as needed. Since 2015, the patient has been undergoing an outpatient behavioral therapy treatment, without achieving complete remission of the OCD so far.

While there were no relevant diseases in the medical anamnesis, the family history revealed that his mother had been diagnosed with schizophrenia and his father had a history of alcohol addiction.

After the initial presentation in our outpatient clinic (December 2017), detailed diagnostic tests were performed, including the Diagnostic Interview for ADHD in adults (DIVA) and ADHD-specific questionnaires [Conners Adult ADHD Rating Scales (CAARS)—Self-Report: Long Version (18), Wender Utah Rating Scale (WURS), and Adult ADHD—Self-Report Scale (ADHD-SB)] as well as other questionnaires (e.g., Personality Styles and Disorder Inventory). The subjective assessment of ADHD-relevant symptoms was clearly significant in terms of inattention and hyperactivity, as well as temperament, affective instability, emotional overreaction, and impulsiveness. The CAARS revealed an ADHD index in percentile rank of 88, a DSM-IV Inattentive symptom scale in percentile rank of 98, a DSM-IV Hyperactive–Impulsive scale in percentile rank of 86, and a DSM-IV ADHD Symptoms Total in percentile rank of 96 (see Table 2). Available school reports were also reviewed: in primary school reports, the patient was described as an eager and endeavored student, who was partly distracted and showed fluctuations in cooperation with other students. A somewhat unfriendly behavior toward classmates was also reported. These descriptions were in accordance with the self-report of the patient and indicate the presence of ADHD in childhood. The available findings as well as the biographical and current anamnesis strongly suggested the diagnosis of ADHD in adulthood.

**Table 2:** The patient's scores on CAARS (in percentile rank) and Y-BOCS.

	**CAARS**	**Y-BOCS**
Diagnostic stage, before ADHD-specific treatment (medication: paroxetine and quetiapine)	DSM-I = 98 DSM-Hy/I = 86 DSM-Total = 96 ADHD-Index = 88	Symptom Checklist: Obsessions: 7/Compulsions: 7 Severity scale: Obsessions: 8/Compulsions: 10
At the end of the first inpatient treatment (medication: ER MPH and sertraline)	DSM-I = 10 DSM-Hy/I = 14 DSM-Total = 10 ADHD-Index = 5	Symptom checklist: Obsessions: 1/Compulsions: 1 Severity scale: Obsessions: 5/Compulsions: 2
During the second inpatient treatment (medication: sertraline, quetiapine, onset of ER MPH treatment after 14 days of atomoxetine intake)	DSM-I = 54 DSM-Hy/I = 82 DSM-Total = 69 ADHD-Index = 76	Symptom checklist: Obsessions: 4/Compulsions: 4 Severity scale: Obsessions: 11/Compulsions: 9
After discharge from second inpatient treatment (medication: ER MPH, sertraline and quetiapine)	DSM-I = 38 DSM-Hy/I = 35 DSM-Total = 35 ADHD-Index = 42	Symptom checklist: Obsessions: 2/Compulsions: 4 Severity scale: Obsessions: 10/Compulsions: 8

*ADHD, attention deficit hyperactivity disorder; ER MPH, extended-release methylphenidate; CAARS, Conners adult ADHD rating scales; DSM-I, DSM-IV inattentive symptoms; DSM-Hy/I, DSM-IV hyperactive–impulsive symptoms; DSM-Total, DSM-IV ADHD symptoms total; Y-BOCS, yale–brown obsessive compulsive scale*.

Due to the complex comorbidity of psychiatric illnesses, the patient was admitted to our inpatient unit in January 2018 for medication adjustment. At that time, the Yale–Brown Obsessive Compulsive Scale (Y-BOCS) (19) was performed to assess the severity of the OCD symptoms. Concerning the last 7 days, the patient affirmed seven out of 37 typical obsessive thoughts and seven of 21 typical compulsive behaviors. In the severity rating, the patient reached a total score of 18 points, of which eight points were scored in the obsessive thoughts scale and 10 points were on the compulsive behavior scale. The laboratory tests showed a mild folic acid deficiency, which was substituted accordingly. Electrocardiography, electroencephalography, as well as magnetic resonance imaging of the brain showed no abnormal findings.

In accordance with existing literature, we switched the medication from paroxetine 30 mg to sertraline 50 mg/day because of the lack of therapy response to paroxetine treatment for many years (20, 21). A psychostimulant treatment with extended-release methylphenidate (ER MPH) was initiated. ER MPH was gradually dosed up to 30 mg/day. Under this medication, not only the ADHD symptoms but also his OCD symptoms improved, so that sertraline could subsequently be reduced to 25 mg/day. At this time, the patient stated that his OCD had almost completely disappeared and that the time he spent with obsessive thoughts and compulsive actions had decreased severely. Furthermore, he felt more balanced and reported that he did not get into conflicts so quickly anymore. As the restlessness decreased, quetiapine could also be reduced and eventually stopped.

One day before discharge (after 42 days on board), Y-BOCS and CAARS were applied again. The patient reported observing one out of 37 typical obsessive thoughts and one of 21 typical compulsive behaviors in the last 7 days. In the severity rating, the patient reached a total score of seven points (five points for obsessive thoughts and two points for compulsive behavior). The CAARS resulted in an ADHD index in percentile rank of 5, a DSM-IV Inattentive symptom scale in percentile rank of 10, a DSM-IV Hyperactive–Impulsive symptom scale in percentile rank of 14, and a DSM-IV ADHD Symptoms Total in percentile rank of 10 (see Table 2). The medication at discharge consisted of ER MPH 30 mg/day and sertraline 25 mg/day.

After discharge, the patient attended our ADHD outpatient clinic for regular follow-ups. On his first visit (1 day after the discharge), he reported a good response to the medical therapy with ER MPH and assured that he did not notice any side effects. He expressed the wish to increase the sertraline dose from 25 to 37.5 mg/day. In the following visit after 26 days, the patient reported unspecific anxiety and panic attacks and claimed to have reduced ER MPH to 10 mg on his own responsibility after having read the package leaflet and worrying about potential side effects. Thus, the remaining medication consisted of sertraline 50 mg/day and quetiapine 25 mg/day, which he started again without a consultation with our outpatient clinic.

In March 2018, a month later after the discharge, a second inpatient admission was initiated after an emergency contact of the patient with the ward. He described an increase in obsessive–compulsive symptoms and restlessness and reported that he suffered from panic attacks and sleep disorders and that he lost his appetite. The patient observed severe mood swings and distrust toward other people. The medication at administration consisted of ER MPH 10 mg/day, sertraline 37.5 mg/day, and quetiapine 25 mg as needed. However, he reported that he did not want to continue to take ER MPH. Therefore, therapy with atomoxetine was started as ER MPH was discontinued. Due to the worsened symptomatology, the sertraline dose was increased to 150 mg/day and quetiapine was dosed up to 125 mg/day. However, the OCD symptoms worsened further after the discontinuation of ER MPH despite increasing the doses of sertraline and quetiapine. After weighing up the symptoms before and after treatment with ER MPH, we decided together with the patient to restart the treatment with ER MPH. Physical well-being and a reduction of the OCD and ADHD symptoms were described after switching the medication from atomoxetine to ER MPH. On the first day of the switch, we performed Y-BOCS and CAARS again. For the last 7 days, the patient reported observing four of 37 typical obsessive thoughts and four of 21 typical compulsive behaviors. In the severity rating, the patient reached a total score of 20 points, of which 11 points were on the scale of obsessive thoughts and nine points were on the scale of compulsive behavior. The CAARS showed an ADHD Index in percentile rank of 76, a DSM-IV Inattentive symptom scale in percentile rank of 54, a DSM-IV Hyperactive–Impulsive scale in percentile rank of 82, and a DSM-IV ADHD Symptoms Total in percentile rank of 69 (see Table 2).

**Figure 1:**
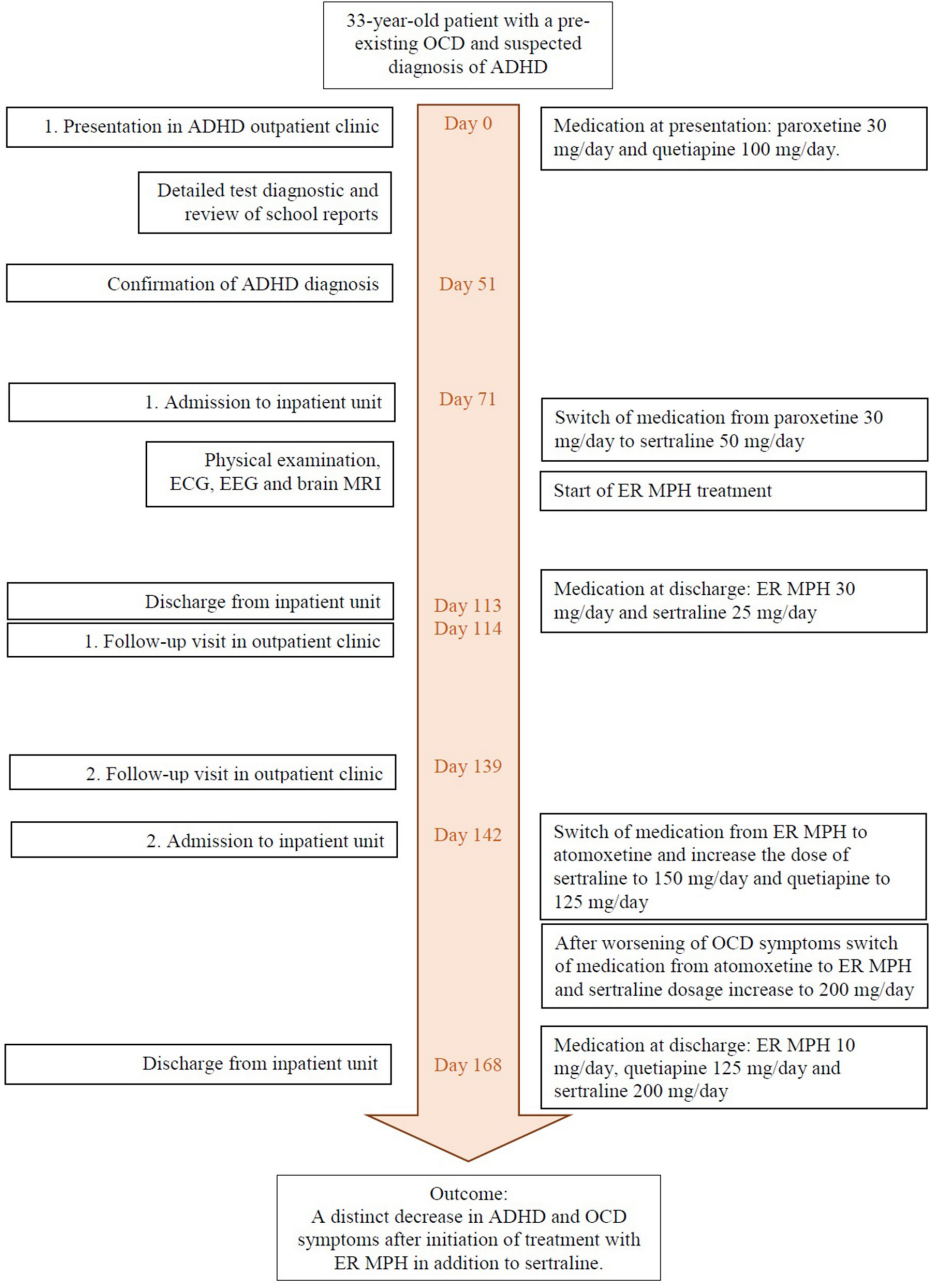
Timeline of events and medication.

An improvement of compulsive thoughts and joyfulness was observed when sertraline was added. The patient was discharged in April 2018 (after 27 days on board) into outpatient care at the ADHS outpatient clinic. Five days after discharge, CAARS and Y-BOCS were performed again: the patient reported observing two of 37 typical obsessive thoughts and four of 21 typical compulsive behaviors within the last 7 days. In the severity rating, the patient reached a total score of 18 points, of which 10 points were on the scale of obsessive thoughts and 8 points were on the scale of compulsive behavior. The CAARS revealed an ADHD Index in percentile rank of 42, a DSM-IV Inattentive symptom scale in percentile rank of 38, a DSM-IV Hyperactive–Impulsive scale in percentile rank of 35, and a DSM-IV ADHD Symptoms Total in percentile rank of 35 (see Table 2). Discharge medication consisted of ER MPH 10 mg/day, quetiapine 125 mg/day, and sertraline 200 mg per/day. A timeline of this case presentation is shown in Figure 1.

## Discussion and Conclusions

In this case report, we present a case of successful treatment with psychostimulants in an adult patient with ADHD and comorbid OCD. Due to the late diagnosis of ADHD (in addition to an apparent misdiagnosis of schizophrenia and personality disorder), no effective treatment was initiated in his early life, resulting in an impacted quality of life up to now. After diagnosing ADHD, we treated the patient with ER MPH in addition to antidepressants for OCD treatment and observed that the adjunctive use of ER MPH resulted in enhanced treatment response. Contrary to reports in the literature, treatment with a stimulant did not cause a worsening of the OCD symptoms. Rather, the patient reported a severe decrease in OCD symptoms, which was also observable by the treatment team. A second administration was necessary due to a worsening of the OCD and ADHD symptoms occurring after the patient had reduced the dose of ER MPH on his own, because he was worried about side effects, which he had never actually experienced during the inpatient treatment. This case highlights the importance of frequent reassessment of comorbid conditions in the case of low treatment response to serotonin reuptake inhibitors and psychotherapy in patients with OCD. Untreated ADHD as a comorbid condition to OCD may reduce the treatment response on the OCD, as shown in previous studies (22).

Recognizing ADHD and OCD comorbidity is important for the clinical course of these disorders considering that the onset of OCD is significantly higher in adults with childhood ADHD symptoms and that the comorbidity is associated with more severe OCD symptoms and their persistence (23, 24). Despite the increasing awareness and interest in ADHD, many affected adults are still underdiagnosed and untreated (25). The overlap of ADHD symptoms with several other psychiatric disorders, including mood disorders, substance abuse, and anxiety, and the high incidence of comorbid psychiatric conditions are probable reasons for the high number of missed ADHD diagnoses in adults (1, 4).

On the basis of neuroimaging findings, structural and functional abnormalities in ADHD and OCD have been reported (26). A shared dysfunction in the mesial frontal cortex has been shown in patients with ADHD and OCD. On the other hand, disorder-specific dysfunctions were found in the caudate, cingulate, and parietal brain regions in patients with ADHD and in the lateral prefrontal cortex in OCD patients (27). Furthermore, fronto-striatal hypoactivity was observed in ADHD, whereas OCD shows fronto-striatal hyperactivity, which is also associated positively with symptom severity (10). Regarding structural abnormalities, a recent meta-analysis reported that patients with OCD have larger insular–striatal regions, whereas patients with ADHS have smaller ventrolateral prefrontal/insular–striatal regions (28). Nonetheless, apart from these disorder-specific abnormalities, both disorders show a similar neuropsychological impairment in executive functions.

Despite the high prevalence of OCD and ADHD comorbidity, only a few reports on the treatment of this comorbidity exist. Most of these studies were performed in child and adolescent populations, and as far as we know, only one was conducted in an adult population (14). Some of the case reports described obsessive–compulsive symptoms as a side effect of MPH treatment in patients with ADHD (12–14, 29–32). However, a few studies also described a decrease of the obsessive–compulsive symptoms with MPH treatment (15, 16). The latter results are in line with our findings. Still, there are no longitudinal and clinical controlled trials investigating the effect of MPH on the treatment of ADHD and OCD comorbidity. Although this case presentation is the first published report of a positive effect of ER MPH for the treatment of ADHD and OCD comorbidity in an adult patient, it also has certain limitations. This case report describes only one patient and a psychostimulant treatment with ER MPH in addition to the therapy with sertraline and quetiapine instead of a monotherapy. Also, it cannot be determined whether the patient took his medication regularly as prescribed after the first discharge.

The present case report highlights that treatment with psychostimulants in addition to a serotonin reuptake inhibitor can improve the obsessive–compulsive symptoms as well as the ADHD-specific symptoms in patients with ADHD and OCD comorbidity. Still, the treatment of this comorbidity remains challenging. Underdetection, misdiagnosis, as well as delay in the diagnosis of this comorbidity may cause a reduction in quality of life and low treatment response. Treating both disorders concurrently may help to decrease the symptom severity of both conditions. Monitoring the progress may also support the treatment process, allowing improvement of the treatment compliance as well as observing side effects. Yet, longitudinal and clinical controlled trials are needed to gain more information about the treatment of this comorbidity and to observe the treatment response longitudinally.

## Data Availability Statement

The data analyzed in this study is subject to the following licenses/restrictions: identifying/confidential patient data cannot be shared. Requests to access the data should be directed to the corresponding author.

## Ethics Statement

Written informed consent was obtained from the individual(s) for the publication of any potentially identifiable images or data included in this article.

## Author Contributions

ED-S and MS were the main authors of the manuscript. ED-S performed the literature research on the comorbidity of ADHD and OCD. Both authors participated substantially in the writing and editing of the final manuscript.

## Conflict of Interest

MS has received speaker fees from Lilly, Medice Arzneimitte Pütter GmbH & Co. KG and Servier and was an advisory board member for Shire/Takeda. The remaining author declares that the research was conducted in the absence of any commercial or financial relationships that could be construed as a potential conflict of interest.
