# Supplementary material for: Case Report: Treatment of a Comorbid Attention Deficit Hyperactivity Disorder and Obsessive–Compulsive Disorder With Psychostimulants
Source: Front Psychiatry. 2021 May 12;12:649833. doi: 10.3389/fpsyt.2021.649833 (PMC8149730; doi:10.3389/fpsyt.2021.649833)
Supplement: Supplementary file 1 [file Data_Sheet_1.pdf]

CARE case report guidelines

CARE Checklist of information to include when writing a case report

(cc) BY-NC-ND

Topic	Item	Checklist item description	Reported on Line
Title	1	The diagnosis or intervention of primary focus followed by the words "case report"	P 1
Key Words	2	2 to 5 key words that identify diagnoses or interventions in this case report, including "case report"	L 14-15
Abstract (no references)	3a	Introduction: What is unique about this case and what does it add to the scientific literature?	L 18-25
	3b	Main symptoms and/or important clinical findings	L 27-30
	3c	The main diagnoses, therapeutic interventions, and outcomes	L 30-38
	3d	Conclusion—What is the main "take-away" lesson(s) from this case?	L 39-45
Introduction	4	One or two paragraphs summarizing why this case is unique (**may include references**)	L 46-81
Patient Information	5a	De-identified patient specific information.	L 84
	5b	Primary concerns and symptoms of the patient.	L 87-116,T1
	5c	Medical, family, and psycho-social history including relevant genetic information	L 132-154
	5d	Relevant past interventions with outcomes	L 132-150
Clinical Findings	6	Describe significant physical examination (PE) and important clinical findings.	L 154-169,172-181
Timeline	7	Historical and current information from this episode of care organized as a timeline	L 244, F1
Diagnostic Assessment	8a	Diagnostic testing (such as PE, laboratory testing, imaging, surveys).	L 154-169,172-181
	8b	Diagnostic challenges (such as access to testing, financial, or cultural)	-
	8c	Diagnosis (including other diagnoses considered)	L 104,168-169
	8d	Prognosis (such as staging in oncology) where applicable	-
Therapeutic Intervention	9a	Types of therapeutic intervention (such as pharmacologic, surgical, preventive, self-care)	L 182-187
	9b	Administration of therapeutic intervention (such as dosage, strength, duration)	L 182-187
	9c	Changes in therapeutic intervention (with rationale)	L 202-205,213-219
Follow-up and Outcomes	10a	Clinician and patient-assessed outcomes (if available)	L 192-198,222-239,T2
	10b	Important follow-up diagnostic and other test results	L 233-239
	10c	Intervention adherence and tolerability (How was this assessed?)	-
	10d	Adverse and unanticipated events	-
Discussion	11a	A scientific discussion of the strengths AND limitations associated with this case report	L 289-301
	11b	Discussion of the relevant medical literature **with references**.	L 282-289
	11c	The scientific rationale for any conclusions (including assessment of possible causes)	L 263-281
	11d	The primary "take-away" lessons of this case report (without references) in a one paragraph conclusion	L 302-311
Patient Perspective	12	The patient should share their perspective in one to two paragraphs on the treatment(s) they received	-
Informed Consent	13	Did the patient give informed consent? Please provide if requested	Yes ☒ No ☐
